# From Li-Po to Na-Po technology: functionalized polymer enables fast charging

**DOI:** 10.1093/nsr/nwag122

**Published:** 2026-03-03

**Authors:** Xuhui Yao, Pierre Kubiak

**Affiliations:** National Physical Laboratory, UK; National Physical Laboratory, UK

*“Hence you observe that sodium chloride is encountered there in significant proportions. Now then, it’s this sodium that I extract from salt water and with which I compose my electric cells.”*

*Jules Verne’s “Twenty Thousand Leagues Under the Sea”*

Just like the Nautilus, a highly advanced submarine powered by sodium technology across vast distances, as envisioned by French writer Jules Verne a century and a half ago, sodium-ion battery (SIB) technology is now being transitioned from conceptual frameworks to initial deployment in low-speed electric vehicles [1]. While the intrinsic electrochemical metrics of SIBs may not appear to outperform lithium-ion batteries, sodium technologies may offer a compelling paradigm for sustainable energy storage due to the global abundance of precursor materials, especially if breakthroughs are achieved in the extreme fast-charging (XFC) frontier [2].

Polymer materials feature a tunable spectrum of physicochemical properties, which have broader exposure in various forms in contemporary batteries, from separators and electrolytes to binders [3]. For example, polymer electrolytes can be engineered to achieve high ion transference numbers, effectively mitigating polarization at high current rates [4]. Therefore, the strategic integration of polymers to optimize electrode or electrolyte interfaces has emerged as a pivotal research hotspot for enabling XFC capabilities in practical SIB applications.

**Figure 1.**
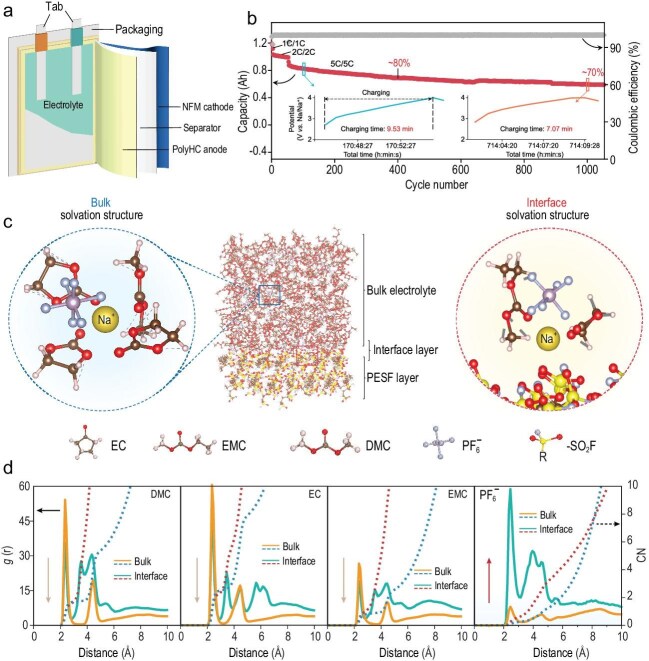
(a) Schematic illustration of the sodium-ion pouch cell architecture. (b) Long-term cycling stability and capacity retention of the pouch cell evaluated at different rates; insets depict the time–voltage profiles during fast charging. (c) Molecular dynamics (MD) simulation snapshots of the polymer layer within a 1 M NaPF_6_ in ethylene carbonate/ethyl methyl carbonate/dimethyl carbonate (EC/EMC/DMC) electrolyte, illustrating representative solvation configurations in both the bulk electrolyte and the interfacial regions. (d) Quantitative spatial distribution of electrolyte species (solvent molecules and anions) derived from MD trajectories, highlighting a higher PF_6_^−^ profile at the PolyHC interface relative to the bulk electrolyte phase. Adapted with permission from Sun *et al*. [5].

In a recent study published in *National Science Review*, Sun *et al.* [5] proposed an innovative interface engineering strategy utilizing polymer material with strongly polar functional groups to stabilize solid-electrolyte interphase (SEI) on the surface of the negative electrode in SIBs. By employing ethylenesulfonyl fluoride (ESF) molecules, the researchers facilitated a 4 nm functionalized polymer *in-situ* coating on a hard carbon electrode (PolyHC). The success was demonstrated in a 1.2 Ah sodium-ion pouch cell with PolyHC as negative electrode and NaNi_1/3_Fe_1/3_Mn_1/3_O_2_ as positive electrode, achieving an energy density of 151 Wh kg^−1^ with the power capability to charge in less than 10 min to over 80% capacity (Fig. 1a and b).

The fundamental advancement lies in a robust SEI, promoted by the enrichment of PF_6_^−^ anions within the Helmholtz layer through highly polar –SO_2_F groups (Fig. 1c and d). By direct observation and characterization of the SEI, the researchers found that introducing ESF polymer can reduce the average SEI thickness from 25 to 5 nm, and such a thin SEI remains consistent and dense over cycling. Surface chemical analysis confirmed a high concentration of NaF and stability of the surface polymer structure. Electrochemical tests revealed lower interfacial impedance, which manifested as enhanced rate capability and cycling reversibility.

As demonstrated by Sun *et al.* [5], polymer interface engineering, a flexible, creative and versatile tool, is showing amazing promise in tackling challenges in battery development, which are well positioned to complement or even outperform conventional inorganic or structural engineering approaches. Looking forward, rational molecular design and multifunctional integration will be key research directions. We believe that some of these innovative achievements will eventually enter people’s daily lives, providing low-cost, high-safety and fast-charging battery energy storage systems.
